# Histopathologic Study of the Effects of Surgically Applied Cryoanalgesia on Intercostal Nerves in a Live Porcine Model

**DOI:** 10.3390/jcm13113304

**Published:** 2024-06-04

**Authors:** Julian Guitron

**Affiliations:** 1 General Thoracic Surgery, The Christ Hospital Health Network, Cincinnati, OH 45219, USA; julian.guitron@thechristhospital.com; Tel.: +1-513-262-2119; 2 Department of Thoracic Surgery, College of Medicine, University of Cincinnati, Cincinnati, OH 45267, USA

**Keywords:** cryoanalgesia, histology, intercostal nerve, pain control

## Abstract

**Background/Objectives**: The aim of this study was to establish a histologic baseline for cryoanalgesia of 2 min duration and evaluate the effects of different freeze durations. **Methods**: A porcine model was used in which the application of bilateral cryoanalgesia from intercostal spaces T3–T7 was completed via partial median sternotomy. The animals were kept alive for 7 days and the ribcages were sent to a specialized center for histopathologic analysis of the freezing injury. **Results**: Forty freezing lesions were completed and analyzed histologically. Thirty-eight (95%) of the cryo-lesions presented 100% nerve fiber degeneration at or distal to the ablation site, with preservation of the perineural connective tissue, as intended. The two unaffected nerves were found to be physically located outside of the freezing area. **Conclusions**: The complete axonal degeneration with preservation of the perineural tissue opens the possibility to shorter freezing times than the recommended 2 min. Visualization of the nerve and positioning of the probe is important in ensuring the proper effect on the nerve. This histologic analysis confirms the process triggered by cryoanalgesia that, until now, had only been assumed.

## 1. Introduction

Pain control after thoracotomy and sternotomy procedures remains a major challenge, with well-known morbidity and mortality implications. However, even sub-centimeter intercostal incisions require a robust multi-modality strategy to achieve adequate levels of pain control. It is estimated that 14% of thoracotomies, 9% of thoracoscopies, and 5% of sternotomies result in narcotic dependance, contributing to the opioid epidemic in the US [1,2]. It is now well documented that a multi-modality pain control strategy is required to optimize outcomes [3]. The use of local anesthetic injections into the intercostal nerves achieves excellent pain control, but the relief is only short-lived. Additional injections are not practical and are rarely offered. Freezing temperatures applied locally have been used for hundreds of years to control pain, providing local analgesia. However, it was not until Cooper in 1962 [4] and Amoils in 1967 [5] that technology allowed for the practical application of controlled and reproducible freezing temperatures of tissue. In 1976, Lloyd and his group specifically described the application of freezing temperature directly to nerves in a reversible way, coining the term “cryoanalgesia” to describe the technique [6].

Intercostal nerve cryoanalgesia is a procedure used to temporarily block nerve conduction along peripheral nerve pathways for pain management, similar to the effects of local anesthetics. The application of freezing temperature results in disruption of the microvasculature that supports the nerve fibers [7]. This disruption stops the conduction of sensory signals back to the central nervous system. Seddon, and later Sunderland, classified the degrees of nerve injury which are of major relevance as it is essential that the disruption be only temporary, with later studies correlating the degree of nerve injury with tissue temperature (Table 1) [8]. At temperatures between −20 °C and −100 °C, axonotmesis occurs, inducing Wallerian degeneration and limiting the analgesia as temporary. During Wallerian degeneration, the surrounding layers of connective tissue (epineurium, perineurium, and endoneurium) are preserved, despite nerve axon injury. The axon degenerates distal from the point of nerve injury and regenerates using the intact connective tissues as a “scaffold” for regrowth. Axonotmesis (Seddon’s classification of nerve injury) with Wallerian degeneration is the goal of cryoanalgesia, blocking signal transmission along the nerve for several weeks or months (depending on the length of the distal axon), as the axon regenerates at a rate of 1–2 mm per day [9]. Sunderland’s equivalent classification is known as a Grade II nerve injury. The result is blockage of pain sensation while the patient recovers post-operatively. Of note, the intercostal vein and artery, part of the neurovascular bundle, are spared from permanent damage as the perivasculature architecture is preserved [10]. Temperatures below −100 °C may cause irreversible injury to the nerve (neurotmesis), inhibiting axonal regrowth [10]. Thus, nitrous oxide and carbon dioxide are most frequently used for cryoanalgesia, as these two gases become solid below their boiling points (−89 °C and −78 °C at 1 ATM), and therefore inherently stay within the desired −20 °C to −100 °C range for axonotmesis [11].

Expert recommendations call for a multimodal, opioid-sparing, pain management strategy as part of enhanced recovery after surgery (ERAS) strategy [3]. Intercostal nerve cryoanalgesia can play a significant role as an adjunctive therapy in this multimodal, non-pharmacological strategy. Historically, published efficacy and side effects have varied significantly, most likely given the variability in techniques, including the freeze duration and devices used. In this study, we aim to provide histological analysis of the effects of cryoanalgesia to the intercostal nerves by applying a commercially available cryoprobe indicated for this purpose to the intercostal nerves of anesthetized pigs intrathoracically via partial median sternotomy. We seek to contribute to the standardization of the technique that has been extremely variable in previous reports through the establishment of a histological baseline for the currently adopted practice of a 2 min freeze and evaluate the efficacy of different freeze durations.

## 2. Materials and Methods

**Figure 1:**
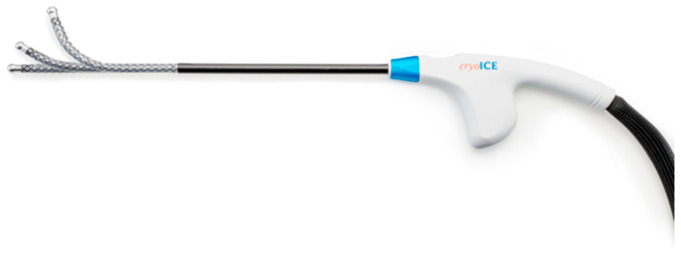
cryoSPHERE probe (AtriCure Inc., Mason, OH, USA).

Study Device: The present study utilized a commercially available cryoprobe (cryoICE cryoSPHERE device [AtriCure, Inc., Mason, OH, USA]) administering a flow of nitrous oxide. The gas expands as it passes through the probe’s internal nozzle (inner tube) into lower pressure within the probe tip (outer tube). The rapid pressure change cools the probe tip to temperatures as cold as the boiling point of the cryogen (−89 °C), although in practicality, presents temperatures that reliably fluctuate between −60 and −70 °C on the exterior surface of the probe tip. The tip of the probe is 8 mm in diameter and made of aluminum, which is highly conductive. Coupled with the high work capacity of nitrous oxide, the probe develops an ice ball when the probe tip is applied to tissue. At the end of the freezing, the probe actively defrosts, allowing for the probe tip to be immediately detached from tissue, leaving the ice ball behind. This facilitates a slow thaw of the ice ball and therefore a longer therapeutic effect. The gas vents back to the cryo-console (ACM, AtriCure, Inc.) through the outer tube, creating a closed system so that no cryogen contacts the patient. The probe features a spherical ball tip, optimized to fit within the intercostal space from various angles of approach, and a malleable shaft to accommodate varying anatomy/port placement while incorporating enough shaft rigidity for steady pressure to be maintained on the target site (Figure 1).

Refs. [7,8,9,10] Study Design: All experimental procedures were reviewed and approved by the IACUC at the Minimally Invasive Training Center, TriHealth on 17 February 2021, under protocol #20-011. The number of animals involved in the experiment was kept to a minimum. Four healthy Yorkshire pigs, weighing 70.8 to 74.8 kg, were utilized across two phases of the study. The first phase of the study used one pig to establish a histological baseline for 2 min freezing of the intercostal nerves. The second phase of the study used three pigs applying different freeze time durations. The freeze duration was semi-randomized across the right and left T3 through T7 intercostal spaces of the four pigs, resulting in forty separate intercostal nerve cryoablations. 

The animals were prepped for surgery by a veterinarian or trained veterinarian technician following routine preoperative protocols. Anesthesia was administered, and the animal was put on a ventilator. The animals were premedicated intramuscularly with buprenorphine hydrochloride 0.05 mg/kg for intra-operative pain and anesthetized intramuscularly with TXK (dosage 1.0 mg/lb ketamine HCl plus 1.0 mg/lb xylazine plus 2.0 mg/lb telazol). The anesthesia was supplemented, over the course of the operative procedure, with isoflurane 1–5% (600 mL to 1 L/min of O_2_ flow) to effect. Continuous monitoring throughout the procedure consisted of ECG, arterial blood pressure from a peripheral vessel, and routine blood gas analyses. 

In preparation for the sternotomy incision, the hair was removed from the animal’s chest area with a razor. Target areas were scrubbed with appropriate antiseptic solutions prior to surgical intervention. The animal was then positioned on the surgical table in dorsal recumbency. A nerve block, Exparel or bupivacaine, was used at the incision site prior to the incision being made. The surgeon then created a partial median sternotomy to gain access to the thoracic cavity.

After the partial median sternotomy, the pleural spaces were entered anteriorly through the parietal pleura, the right and left third through seventh intercostal spaces were marked with a sub-pleural endoscopic marker adjacent to the intended locations of the cryoablation sites, at least 4 cm away from the spine. Specifically, sites were marked using an endoscopic marker (Black Eye™ Endoscopic Marker) and spinal needle (i.e., Quincke type point), which is a common clinical practice used to mark gastrointestinal lesions. The tattoos were created by injecting a small bolus of dye onto the sterile needle tip and then the needle tip inserted sub-plurally at the desired marking location. A total of two small dots were created approximately 8–10 mm apart on the rib bone superior (cranial) to the intended freeze location. A control (sham) site was also marked on the right 8th intercostal space of each animal.

The animal was then subjected to cryoanalgesia (cryoNB) therapy with cryoablations completed intrathoracically on intercostal nerve bundles, one per intercostal space between spaces 3 through 7 bilaterally. These ablations were completed using the cryoSPHERE device with the ACM cryo console. Cryoablations were performed on the superior aspect of each intercostal space, adjacent to each marked site. The ball tip of the probe was pressed into the intercostal space under direct visualization; no dissection was performed to expose the nerve. The pressure of the probe applied to each intercostal site was controlled via a force gage added to the probe. Freeze duration was controlled by the cryo console following a semi-randomized test matrix. The freeze duration was set on the ACM prior to the start of the freezing, with the timer beginning initiated when the probe temperature reached −40 °C or below. The operating temperature of the probe was −68 °C ± 1 °C. A total of five different durations (30, 60, 90, 120, and 180 s) were evaluated in the study with a minimum of six samples per each freeze duration. The order of ablations was determined so that at least one sample per freeze duration would be completed on each intercostal space throughout the duration of the study (i.e., #3, 4, 5, 6, and 7—Left or Right). The order was chosen so that samples per freeze duration were spread out equally between the animal models to account for animal model variation. Device performance was recorded during each freeze. No freezing was applied to the control (sham) site of each animal. 

To close out the procedure, a layer suturing technique was used to close the incision sites and chest. The animals were then recovered and returned for post-operative care and observations. After recovery from the procedure all animals were allowed to survive 7 days. Each animal received buprenorphine hydrochloride 0.05 mg/kg intramuscularly every 8 h for 2–3 days post-operatively or a one-time dose of sustained-release buprenorphine sustained 0.12 mg/kg–0.24 mg/kg intramuscularly for post-operative pain. Additionally, animals received Cerenia–Maropitant citrate 1 mg/kg intramuscularly once a day for 3 days as an anti-inflammatory. On post-operative day 7, the animals were humanely euthanized, and rib sections collected and placed in 10% neutral-buffered formalin in heat-sealed bags for histological processing and evaluation (StageBio, Frederick, MD, USA). Each rib section with adjacent intercostal tissue was trimmed to isolate a single rib/intercostal site. This included freeze sites T3 to T7, bilaterally, as well as the control site on the right 8th intercostal space of each animal. The ribs and corresponding intercostal spaces were labeled, adding suture or tissue dye to clearly label the proximal vs. distal sites to ablation.

Refs. [9,10,11,12,13] Histological processing and analysis [14]: Cross-section slices of the tissue were taken at the level of the treatment site, as well as 2.5 cm proximal and distal to the center of the ablation site and embedded in a paraffin wax. One longitudinal nerve segment was submitted for paraffin embedding and a cross-section was submitted for post-fixation in osmium tetroxide and plastic resin embedding at each treatment site, as well as 2.5 cm proximal and distal to the center of the ablation site. All paraffin-embedded tissues were stained with hematoxylin and eosin (H&E). Resin-embedded tissues were stained with toluidine blue (TB). The tissue and nerve response to cryoablation, including nerve fiber/axon degeneration and damage to surrounding tissue and vascular bundle were semi-quantitatively evaluated by a contract research organization veterinary pathologist with Stage Bio. Quantitative measurements using ImageJ software were also taken (Phase 2 only) from the treatment site paraffin-embedded cross-section, including the affected zone depth, depth of the nerve, and distance of the nerve from the rib. The histological criterium for successful cryoablation was defined as complete (100%) nerve fiber degeneration (axonotmesis, Grade II nerve injury) at or distal to the ablation site. 

## 3. Results

**Figure 2:**
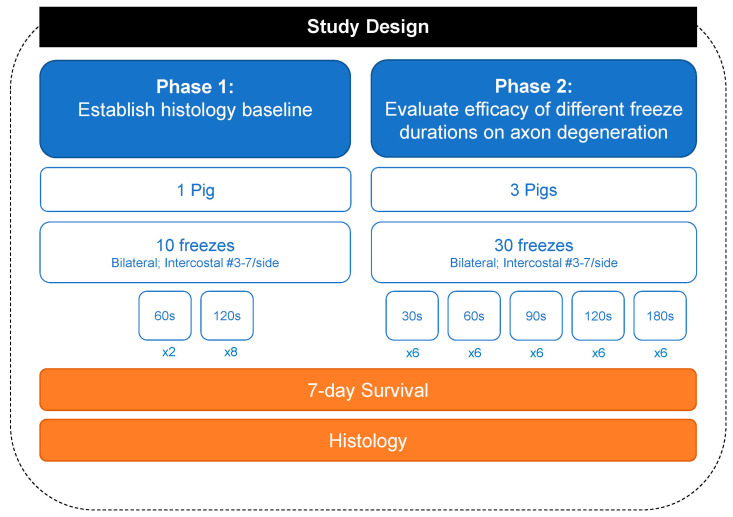
Flowchart summarizing the 40 freezing lesions.

**Figure 3:**
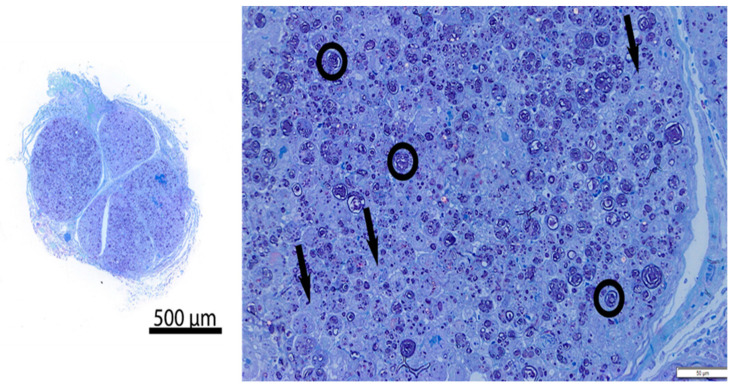
Representative resin cross-section of intercostal nerve at ablation site (Pig 4, Left intercostal space 5, freeze duration = 90 s). **Left**—overview; **Right**—high magnification image. There is diffuse nerve fiber degeneration. The circled representative axons show loss of normal structural axon/myelin ration and fragmentation of myelin. Arrows = vacuolated histiocytes. Compared to control nerve in Figure 4.

**Figure 4:**
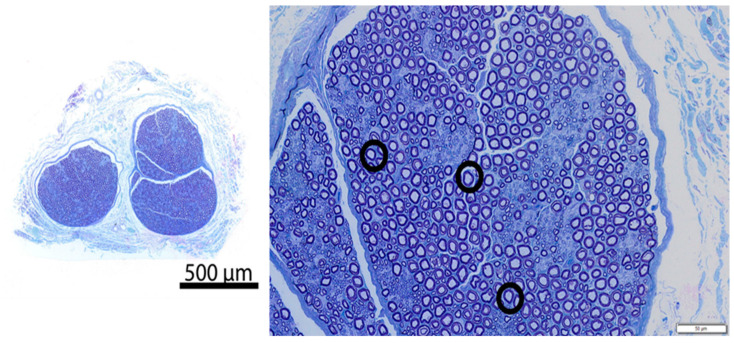
Representative resin cross-section of control intercostal nerve (Pig 4, Right intercostal space 8, no freeze). **Left**—overview; **Right**—high magnification image. The section in the image is morphologically normal. The circled representative axons show normal structural axon/myelin ratio, and both axon and myelin are intact.

**Figure 5:**
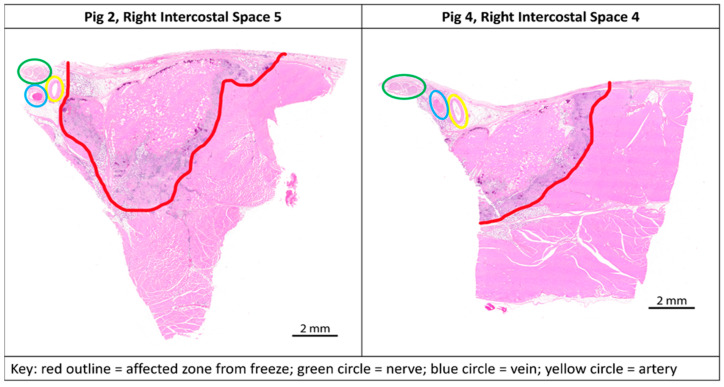
Transverse paraffin-embedded slides; ablation sites of the two outliers from study with anatomical landmarks marked. **Left** (Pig 2, Right intercostal space 5)—nerve (green circle) outside of the affected zone (red outline). **Right** (Pig 4, Right intercostal space 4)—nerve (green circle) on the edge of the affected zone (red outline).

All four animals (Phase 1: 72.5 kg; Phase 2: 72.5 kg, 74.8 kg, 70.7 kg) survived the surgical procedure and the 7-day post-operative period. Ten tissue samples were prepared per animal for a total of 40 samples (Figure 2). Successful cryoablation was found in 95% (38/40) of the samples (Figure 3 and Figure 4). These complete (100%) nerve fiber degeneration results were found at the treatment site across all freeze durations evaluated (30, 60, 90, 120, and 180 s). The two unsuccessful ablation sites were found to be related to probe positioning relative to the nerve (Figure 5). For all samples, the nerve proximal to the ablation site was unaffected, as evidenced by no or minimal nerve degradation in the cross-sections taken 2.5 cm proximal to the center of the ablation site.

**Figure 6:**
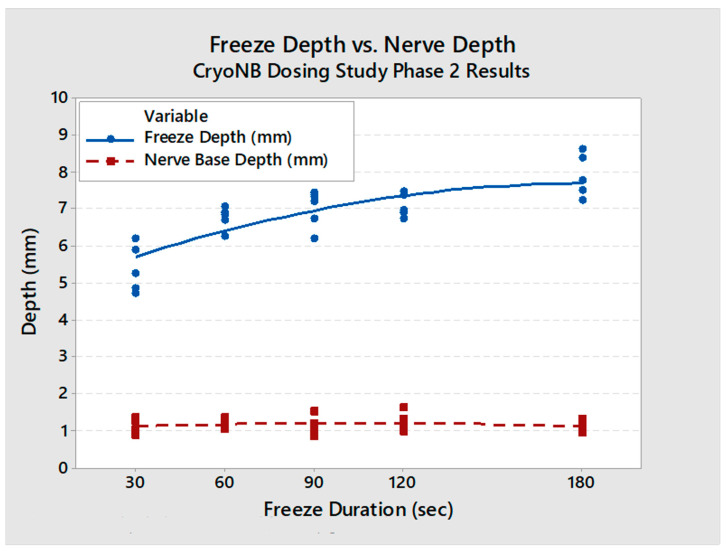
Affected zone depth (ablation depth into soft tissue) and nerve depth vs. freeze duration (with regression equations). The affected zone depth (blue) was found to be deeper than the native nerve depth (red) in all cases.

Changes in surrounding tissues were, as expected, affecting tissues mainly at the level of the ablation sites, and were morphologically consistent with direct freeze effects. Intercostal artery and vein proximal and distal to the ablation sites generally showed no or minimal changes and were interpreted to have been preserved. Ablation-related changes at the level of the ablation site (including endothelial cell loss, necrosis, mural inflammatory cell infiltrates, and thrombosis) were within the expected range of changes in cryo-ablated tissues at 7 days with the vascular architecture preserved. Quantitative measurements found that the affected zone depth increased with freeze duration (30 s: 5.4 ± 0.5 mm; 60 s: 6.2 ± 0.6 mm; 90 s: 6.6 ± 0.8 mm; 120 s: 6.5 ± 0.6 mm; 180 s: 7.6 ± 0.6 mm). At all freeze durations, the freeze depth was deeper than the nerve (Figure 6). Nerve location within the intercostal space of the porcine model varied from 0.7 mm to 6 mm away from the rib (mean distance: 3.8 mm). 

## 4. Discussion

We met our study objectives of establishing a histological baseline for the current recommended practice of a 2 min freeze and evaluating the efficacy of different freeze durations using a consistent technique of probe application. The study was structured with a minimum 7-day survival time to capture Wallerian degeneration [9,15]. A 2 min freeze with the cryoSPHERE probe was histologically shown to result in Wallerian degeneration (axonotmesis, Grade II nerve injury), as evidenced by complete (100%) nerve fiber degeneration at or distal to the ablation site (Figure 3 and Figure 4). The endoneurium, perineurium, and epineurium of the nerves were found to be intact. Similar nerve degeneration results were found at all other freeze durations evaluated (30 s, 60 s, 90 s, and 180 s), except two of the 40 intercostal sites that did not result in successful ablation. On analysis of the histology slides of these two sites, we found that the nerve was located outside of the therapeutic frozen zone or at the margin of the treated zone (Figure 4). The ice ball was the coldest at the surface of the probe tip and got warmer toward the edges. A nerve on the edge of the affected zone may not have been subjected to −20 °C or colder temperature required for therapeutic effect. These two outliers highlight the fact that nerve location varies. In this study, nerve location was found to vary in distance away from the rib in pigs (range: 0.7 mm to 6 mm). The nerve location relative to the rib has also been found to vary in humans [16]. A close approximation of the probe to the nerve is critical for intercostal nerve blockage success and adequate temporary pain relief without damage. Furthermore, reaching optimal ice ball size during cryoanalgesia procedures is important to account for anatomical differences and missing targeted nerve structures. This study used a consistent technique of probe application, including maintaining pressure applied to the intercostal site throughout the freezing, which helps ensure good contact of the ball tip in order to extract heat from the tissue. 

Early studies of intercostal nerve cryoanalgesia have reported mixed results, but also significant variability in the techniques and devices used. This ranges from the different freeze time durations, temperatures applied, number of nerves frozen, and standard of care analgesia studied to the surgical technique in which the parietal pleura is opened, and the nerve dissected and exposed [17,18,19,20,21]. In unsupportive studies, post-operative pain relief and rescue analgesia consumption were equivalent in the cryoanalgesia and control analgesia groups. Cryoanalgesia was reportedly associated with a greater incidence of pain, numbness, and neuropathic pain that persisted longer than 2 months [19,20]. It was unclear whether the potential long-term neuropathic pain occurred because of cryoanalgesia or the surgical procedure in general. In supportive studies, the use of cryoanalgesia has been shown to significantly improve post-operative pain scores [17], significantly reduce the hospital length of stay [22], reduce narcotic requirements [17,18,23], and improve pulmonary mechanics [14,17]. A recent study of pectus excavatum patients has shown encouraging results, with 92.5% of patients discharged to home on post-operative day one when intercostal nerve cryoablation was performed as part of a multimodal pain management plan [24]. Cryoanalgesia adds operative time to the procedure depending on the number of intercostal spaces to be treated. Traditionally, 2 min of freeze time per nerve has been used, adopted from experience with cardiac cryoablation. As mentioned previously, there is significant variability in the times and temperatures used in the current literature. In general, 4–5 intercostal treatments add 10 to 20 min to the procedure. A recent randomized trial, FROST, reported on the effectiveness of the 2 min freeze time utilizing an AtriCure, Inc. cryoprobe [14].

In the present study, we found successful intercostal cryoablation (resulting in Wallerian degeneration) using a consistent technique for intrathoracic cryoanalgesia, for the currently adopted practice of a 2 min freeze and for different freeze durations. 

## 5. Conclusions

The results from this study are specific to AtriCure, Inc cryoICE cryosphere technology, and open the possibility of performing shorter freeze durations to achieve therapeutic effects on the nerve. This study was performed on healthy pigs with minimal adipose tissue present on the pleural surface of the thoracic cavity. In humans, there is significant variability in the amount of adipose tissue present that could impact the affected zone from the freezing. The differences between human anatomy and that of the porcine model require further studies to provide a definitive recommendation regarding the reduction in cryoablation time for human use. Close proximity of the probe to the nerve is critical for intercostal nerve blockage success, but as demonstrated in this study, the parietal pleura can be left intact. Though this study confirmed the presence of axonotmesis after a 7-day survival time, a study with a shorter end point is needed to measure the immediate impact of cryoNB when applied with shorter freeze time durations. In addition, a longer study duration is needed to see the regeneration of the nerves [17], as this study’s end point was not long enough to demonstrate the regeneration of the axons of the nerve. This study suggests that a freeze time of less than 2 min is both feasible and effective with the cryoICE cryosphere probe design, and further research is needed to confirm these results clinically.

## Figures and Tables

**Table 1:** Degree of nerve injury summary, including classifications from Seddon [12] and Sunderland [9], and temperature correlation from later studies [13].

Seddon’s Classification	Sunderland’s Classification	Tissues Injured	Tissues Intact	Temperature	Effect on Nerve Function
Neuropraxia	Grade I	Myelin	Axon, Endoneurium, Perineurium, Epineurium	+10 °C to −20 °C	Minimal histological changes with short recovery
Axonotmesis	Grade II	Myelin, Axon	Endoneurium, Perineurium, Epineurium	−20 °C to −100 °C	Loss of continuity of axon; Wallerian degeneration; temporary disruption of nerve function (recovery time dependent on length of distal axon)
Neurotmesis	Grade III	Myelin, Axon, Endoneurium	Perineurium, Epineurium	Colder than −100 °C	Irreversible injury; low regeneration possibility
	Grade IV	Myelin, Axon, Endoneurium, Perineurium	Epineurium		
	Grade V	Myelin, Axon, Endoneurium, Perineurium, Epineurium	None	N/A—Transection of nerve	

## Data Availability

The original contributions presented in the study are included in the article, and further inquiries can be directed to the corresponding author.
